# The Efficacy of Moxibustion on the Serum Levels of CXCL1 and *β*-EP in Patients with Rheumatoid Arthritis

**DOI:** 10.1155/2021/7466313

**Published:** 2021-10-13

**Authors:** Siyu Tao, Xue Wang, Chenxi Liao, Yan Xiong, Jie Tang, Nannan Jiang, Yuan Li, Xinyue Hu, Rouxian Shuai, Yueyue Wang, Ping Wu

**Affiliations:** ^1^Chengdu University of Traditional Chinese Medicine, Chengdu 610075, Sichuan Province, China; ^2^West China Fourth Hospital, Chengdu 610075, Sichuan Province, China; ^3^Chengdu University of Traditional Chinese Medicine Affiliated Hospital, Chengdu 610075, Sichuan Province, China; ^4^Sichuan Integrative Medicine Hospital, Chengdu 610075, Sichuan Province, China

## Abstract

**Objective:**

This study aims to evaluate the efficacy of moxibustion on joint swelling and pain and the levels of C-X-C motif chemokine ligand 1 (CXCL1), *β*-endorphin (*β*-EP) in serum of rheumatoid arthritis (RA) patients and to investigate the anti-inflammatory and analgesic mechanism of moxibustion on improving RA.

**Methods:**

Sixty-eight patients with RA were randomly and equally classified into the control and treatment groups. The control group was treated with routine drug therapy, while the treatment group received routine drug therapy and moxibustion. Both groups were treated for eight weeks. The symptoms and laboratory indicators of RA patients were compared in the two groups before and after intervention.

**Results:**

Sixty-one patients completed the study: four patients dropped out from the treatment group and three from the control group. Trial endpoints were change (∆) in symptoms, measured by Ritchie's articular index (RAI), swollen joint count (SJC), and laboratory indicators, measured by the level of CXCL1, *β*-EP, tumor necrosis factor-*a* (TNF-*α*), and interleukin-1*β* (IL-1*β*). ∆RAI, ∆SJC, ∆CXCL1, ∆*β*-EP, ∆TNF-*α*, and ∆IL-1*β* in the treatment group were superior to the control group (13.50 [14.50] versus 6.00 [13.00] in ∆RAI, 4.00 [3.00] versus 2.00 [4.00] in ∆SJC, 0.04 ± 0.79 ng/mL versus -0.01 ± 0.86 ng/mL in ∆CXCL1, -2.43 [5.52] pg/mg versus -0.04 [4.09] pg/mg in ∆*β*-EP, 3.45 [5.90] pg/mL versus 1.55 [8.29] pg/mL in ∆TNF-*α*, and 6.15 ± 8.65 pg/mL versus 1.28 ± 8.51 pg/mL in ∆IL-1*β*; all *P* < 0.05).

**Conclusion:**

Moxibustion can improve the joint swelling and pain symptoms in patients with RA, which may be related to the fact that moxibustion can reduce the release of inflammatory factors in patients with RA and downregulate the level of CXCL1 and increase the level of *β*-EP at the same time. This trial is registered with ChiCTR-IOR-17012282.

## 1. Introduction

Rheumatoid arthritis (RA) is a chronic, systemic autoimmune disease characterized by pain, stiffness, swelling, and tenderness of synovial joints, resulting in joint destruction, disability, and decline in quality of life [1]. The global incidence of RA is about 0.5–1.0% [2], and the incidence in China is about 0.5% [3]. The lifetime prevalence rate is as high as 1% in the world. The course of RA can be as long as several decades. Joint swelling and pain caused by persistent inflammation is one of the important factors to reduce the quality of life of RA patients, and it is also a problem that patients urgently want to solve [4].

The swelling and pain of RA is closely related to the continuous release of inflammatory pain-causing substances in joints. Cytokines released by inflammatory cells in joints, such as tumor necrosis factor superfamily, interferon, interleukin, and chemokines, are all important pain-causing substances that promote continuous joint inflammatory response and hyperalgesia in RA patients [5–7]. Chemokines are chemotactic cytokines that regulate the migration of immune cells during various physiological and pathological processes. In the chronic inflammatory process of RA, chemokines are involved in the activation and proliferation of T lymphocytes and angiogenesis by inducing the migration of a large number of inflammatory cells from peripheral blood to the synovium of the joint, thereby destroying the synovium of the joint [6, 8]. C-X-C motif chemokine ligand 1 (CXCL1) is one of the chemokines secreted by fibroblasts in synovium mediated by tumor necrosis factor-*a* (TNF-*α*) and interleukin-1*β* (IL-1*β*). It can promote inflammatory response [9], promote angiogenesis [8], and enhance the sensitivity and excitability of peripheral sensory neurons [10, 11]. However, it is important to note that inflammation not only plays a painful role but also triggers immune cells containing opioid peptides to gather and release endogenous opioid peptide at the inflammatory site, resulting in an analgesic effect [12–14]. *β*-endorphin (*β*-EP), as an endogenous opioid peptide with typical opioid like effects, not only acts on opioid receptors on primary afferent neurons through secretion of immune cells, blocking pain transmission, so as to achieve analgesic effect [15], but also regulates immune function by binding to receptors on immune cells [16, 17]. Therefore, the changes of chemokines and endogenous opioid peptides in vivo are closely related to the disease development and clinical symptoms of RA patients.

RA is considered to be an incurable chronic disease, and modern medicine is the current mainstream treatment. However, the side effects and high cost of long-term medication have brought great trouble to patients' body and mind. In China, moxibustion has a history of thousands of years in the treatment of RA, and its safety, effectiveness, and low price are its core competitiveness to be accepted by patients. Modern studies have found that the efficacy of moxibustion is related to inflammatory factors, signaling pathways, protein expression, and other aspects [18, 19]. However, most of the relevant studies focus on animal studies, while few clinical studies. In recent years, we gradually focus on the clinical mechanism of moxibustion [20, 21]. In this study, the effects of moxibustion on serum levels of CXCL1 and *β*-EP in RA patients were observed to further explore the mechanism of moxibustion in improving joint swelling and pain symptoms in RA patients, so as to provide evidence for the clinical treatment of RA.

## 2. Materials and Methods

### 2.1. Selection Criteria

**Figure 1:**
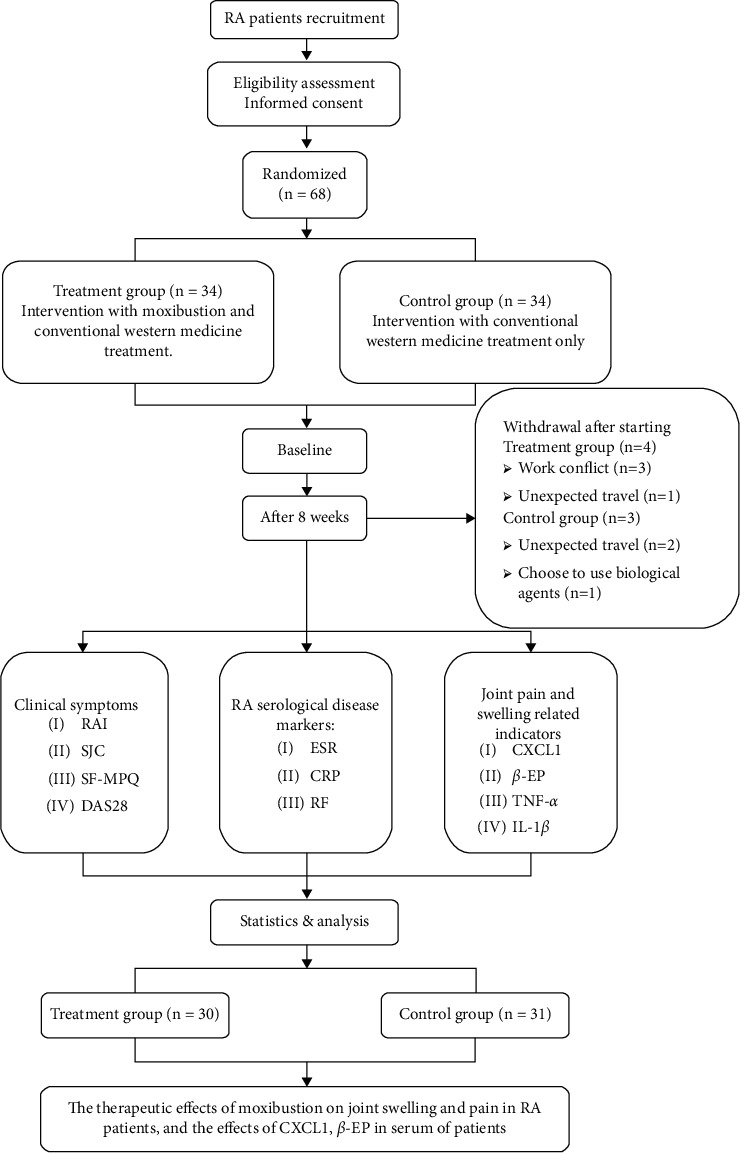
Technology roadmap.

Between July 2018 and January 2020, we recruited 68 patients with RA in Sichuan Provincial Hospital of Traditional Chinese Medicine based on inclusion/exclusion criteria and randomly divided them into treatment group and control group (Figure 1). This study was in line with the Declaration of Helsinki and was approved by Sichuan Provincial Chinese Medicine Regional Ethics Review Committee (no. 2015KL-05). Prior to the start of the study, we had detailed information about the content of the study to all enrolled patients and obtained written informed consent from each participant.

#### 2.1.1. Inclusion Criteria

The patient must meet all of the following conditions:

1. Diagnosed as RA by clinical experts according to the diagnostic criteria revised in 2010 by the American College of Rheumatology (ACR) and the European League Against Rheumatism (EULAR) [1]
2. between 18 and 70 years old (including 18 and 70 years old)
3. DAS28 (disease activity score of 28 joints) score > 3.2
4. Not using any other types of antirheumatoid drugs within half a year
5. The course of disease is more than three months
6. The patient has a clear consciousness and mind and is able to cooperate to complete the test
7. good compliance, voluntarily cooperates with the study, and signs the informed consent

#### 2.1.2. Exclusion Criteria

The patients will be excluded if they meet any of the following conditions:

1. under 18 years old or over 70 years old
2. patients with advanced RA (patients with IV joint function [22])
3. patients with other autoimmune diseases (such as SLE, sicca syndrome, and ankylosing spondylitis)
4. accompanied by communicable disease (such as tuberculosis), malignant tumors, and infectious diseases (such as osteomyelitis)
5. accompanied by serious underlying diseases (such as hypertension, heart function, liver function, and renal insufficiency)
6. patients with mental retardation who could not independently fill in the questionnaire and complete the whole test
7. patients with thrombocytopenia or platelet coagulation dysfunction, allergic constitution, and skin disease patients and patients allergic to moxibustion treatment
8. pregnant and lactating women
9. those who participate in other studies at the same time
10. patients who do not obey the test arrangement and do not complete the test process according to the regulations
11. those who are afraid of moxibustion treatment or are allergic to moxa smoke and many kinds of drugs

#### 2.1.3. Sample Size

According to previous research [21], after moxibustion, the mean pain score of RA patients in the treatment group was 3.32 points and the standard deviation was 1.65 points, while the mean pain score of RA patients in the control group was 4.72 points and the standard deviation was 1.92 points. This study is set up according to the ratio of 1 : 1 to test the level *α* = 0.05, inspection efficiency 1- *β* = 0.90. The sample size was estimated using *t* tests in *G*^*∗*^ power (version 3.1.7, Franz Faul, University Kiel, Germany). Through calculation, the comprehensive effect size is 0.782. It is estimated that the total number of samples required in the treatment group and the control group is 58. The samples are calculated according to the 15% loss rate. The sample size is 68 cases, and there should be no less than 34 cases in each group. In fact, a total of 68 RA subjects who met the criteria were included in this trial, and 61 cases were actually completed (Figure 1).

### 2.2. Random and Blind Method

The random numbers generated by SPSS 26.0 software (SPSS, Inc., Chicago, Illinois, USA) are used, which were annotated on cards that were randomly placed in light-proof, sequentially numbered, and sealed envelopes. The eligible patients were randomly assigned to the treatment group and the control group in a 1 : 1 ratio. The random method is supervised by a special data statistician. The whole study strictly followed the requirements of acupuncture clinical trials. In view of the specificity of moxibustion operation used in the trial, it is easy for patients in the treatment group to know the treatment plan, so it is difficult to meet the standard double-blind requirements. Therefore, blind evaluation and blind statistics were carried out in this experiment. During the whole study process and data analysis, the principle of separation of moxibustion operators, efficacy evaluators, and data statistical analysers were strictly followed.

### 2.3. Interventions

**Figure 2:**
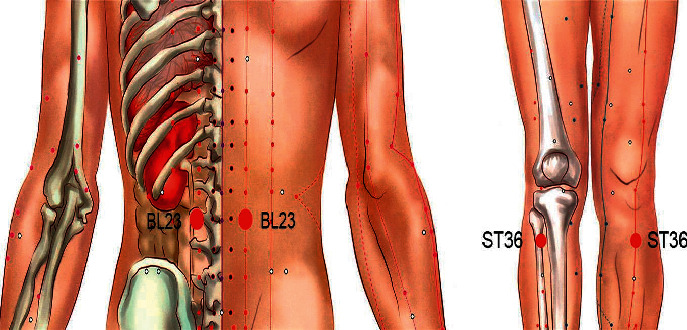
Acupoints used in the trial. The two acupoints are BL23 on the lower back and ST36 on the lateral side of the knee.

All patients in the study were treated with conventional drugs. The treatment regimen was oral methotrexate (7.5 mg, once a week) and folic acid (10 mg, once a week), and moxibustion was added at bilateral Zusanli points (ST36), bilateral Shenshu points (BL23), and A-Shi points in the treatment group (Figure 2).

**Figure 3:**
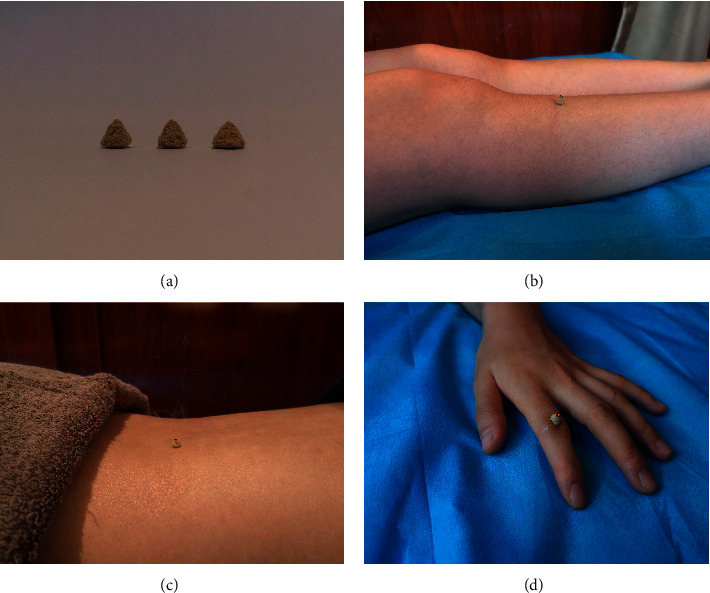
Diagram of the grain-sized moxibustion. (a) Samples of small moxa cones. (b) Patients will undergo treatments at acupoints ST36. (c) Patients will undergo treatments at acupoints BL23. (d) Patients will be treated at A-Shi acupoints.

The selection of acupoints is based on our previous research, which found that moxibustion at ST36, BL23, and A-Shi points can effectively improve the clinical symptoms of RA and the content of related serum markers [20, 21], and according to the State Standard of the People's Republic of China (GB/T123462006). The name and location of the acupoint are shown in Figure 2. The moxibustion method in the experiment is wheat grain moxibustion, which is a moxibustion method of skin contact. Moxibustion physicians will use moxa grass (Nanyang Yile Moxibustion Co., Ltd., China) to process a small moxa cone (about 3-4 mm in diameter and height) (Figure 3(a)), requiring patients to be treated in a comfortable position. The acupuncturist then marks the acupoints with a marker and applies a thin layer of vaseline around the acupoints to help the moxa cone stick. Then, the moxa was placed directly on the acupoint and ignite the top. When the moxa cone burned close to the skin and the patient felt a slight burning sensation, immediately use tweezers to remove moxa cone, replace it, and perform moxibustion again. Seven moxa cones shall be used for moxibustion per acupoint for each time (Figures 3(b)–3(d)). After the previously mentioned operations, the local skin flushing without blistering was taken as the degree. Moxibustion treatment was performed twice a week for four weeks as a course of treatment (a total of two courses).

### 2.4. Outcome Variable

Trial main endpoints were improved in symptoms, measured by the Ritchie's articular index [23] (RAI indicates the degree of joint pain), and the swollen joint count (SJC) and laboratory indicators, measured by the level of CXCL1, *β*-EP, TNF-*α*, and IL-1*β* in serum. As secondary endpoint, changes in the severity of the disease were tested. They were assessed by the simplified McGill pain questionnaire [24] (SF-MPQ: this pain questionnaire is commonly used in clinic to evaluate the pain status of acute or chronic inflammation), disease activity score 26 of 28 joints [25] (DAS28 evaluates RA disease activity), and the level of rheumatoid factor (RF), erythrocyte sedimentation rate (ESR), and C-reactive protein (CRP) in serum.

### 2.5. Specimen Collection

Elbow venous blood samples were collected from the subjects before and after treatment at the laboratory department of Sichuan Provincial Hospital of Traditional Chinese Medicine, respectively. The blood was kept at room temperature for 15 min, centrifuged at 3000xg for 5 min, and stored at − 80°C immediately. After treatment, serum samples were sent to Chengdu Lilai Biomedical Laboratory Center for detection, and the contents of CXCL1, *β*-EP, TNF-*α* and IL-1*β* in serum were detected by enzyme-linked immunosorbent assay (ELISA).

### 2.6. Statistical Analysis

SPSS 26.0 statistical software was used to analyze the data. Chi-square (*χ*^2^) test was used for counting data. For measurement data of normal distribution, paired sample *T* test was used for intragroup comparison, and independent sample *T* test was used for intergroup comparison. For measurement data of nonnormal distribution, Wilcoxon rank sum test was used for intragroup comparison, and Mann Whitney *U* test was used for intergroup comparison. Gaussian distribution values are represented as mean ± standard deviation (SD), and non-Gaussian distribution values are represented as median (first quartile to third quartile). *P* < 0.05 indicated a statistical difference, and *P* < 0.01 indicated a significant difference.

## 3. Results

### 3.1. Baseline Characteristics

In the end, a total of 61 patients completed the study, with four patients dropped from the treatment group and three from the control group (Figure 1). There were a total of 30 patients in the treatment group, including four males and 26 females, with an average age of 53.0 ± 8.80 years and an average course of disease of 10.11 ± 9.02 years. There were 31 patients in the control group, including five males and 26 females, with an average age of 49.39 ± 7.72 years and an average course of disease of 9.13 ± 9.09 years. There was no significant difference in general information between the two groups (*P* > 0.05), and the baseline was balanced and comparable.

The baseline characteristics of clinical symptoms and routine serum indicators of RA in the control and treatment groups are shown in Table 1 (clinical symptoms and routine inspection indexes), and the baseline characteristics of CXCL1, *β*-EP, TNF-*α*, and IL-1*β* are shown in Table 2.

### 3.2. The Joint Swelling and Pain Symptoms and RA Serological Disease

After treatment, RAI, SJC, DAS28, and SF-MPQ in two groups were significantly improved (four indexes in treatment group, *P* < 0.01; for RAI, *P* < 0.01 in control group; the other three, *P* < 0.05). The results showed that the symptoms of joint swelling and pain were significantly improved, and the disease activity was significantly decreased in both groups (SJC and DAS28, *P* < 0.05; SF-MPQ, *P* < 0.01) (Table 1).

After eight weeks of treatment, treatment group (*P* < 0.01) and control group (CRP and RF, *P* < 0.01; ESR: *P* < 0.05), the levels of ESR, CRP, and RF were significantly decreased. However, there was no significant difference in ESR, CRP, and RF between the two groups before and after treatment (*P* > 0.05), indicating that there was no significant difference in the improvement degree of routine serum indexes between the two groups (Table 1).

### 3.3. The Contents of CXCL1, *β*-EP, TNF-*α*, and IL-1*β*

**Figure 4:**
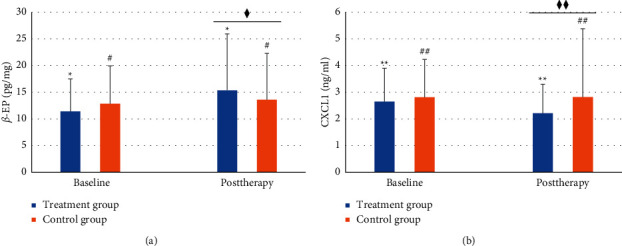
*ß*-EP and CXCL1 contents in the serum of two groups. The content of *ß*-EP, ^*∗*^*P* < 0.01, ^#^*P* > 0.05, ^♦^*P* < 0.05. The content of CXCL1, ^*∗*^^*∗*^*P* < 0.01, ^##^*P* > 0.05, ^♦♦^*P* < 0.01. The data of *β*-EP were expressed as median (first quartile to third quartile), and the data of CXCL1 were expressed as mean (SD).

After treatment, the contents of CXCL1, *β*-EP, TNF-*α*, and IL-1*β* in serum of the subjects in treatment group were significantly decreased (*P* < 0.01). The contents of CXCL1, *β*-EP, TNF-*α*, and IL-1*β* in serum of control group were not significantly changed after treatment (*P* > 0.05). After eight weeks of treatment, the changes of CXCL1, *β*-EP, TNF-*α*, and IL-1*β* in the treatment group were significantly better than those in the control group (Table 2; Figure 4).

In the treatment group, the difference of CXCL1 in serum of the objects was positively correlated with the difference of TNF-*α* and IL-1*β* before and after treatment (*P* < 0.01), and the difference of *β*-EP in serum was negatively correlated with the difference of TNF-*α* and IL-1*β* (*P* > 0.05) (Table 3).

## 4. Discussion

In this study, the effects of moxibustion on the contents of CXCL1, *β*-EP, IL-1*β*, and TNF-*α* in serum of RA patients were observed and compared with conventional drug therapy, to further reveal the mechanism of action of moxibustion in the treatment of RA.

### 4.1. The Efficacy of Moxibustion on Joint Swelling and Pain Symptoms

RA is an autoimmune disease. It is an abnormal function of the immune system that affects the joints leading to persistent joint inflammation, which is characterized by stiffness, swelling, and pain. Unfortunately, there is no radical treatment for RA. Although nonsteroidal anti-inflammatory drugs (NSAIDS), disease modifying antirheumatic drugs (DMARDs), and corticosteroids can be used for controlling, but side effects are obvious. Some patients lose responsiveness after long-term drug treatment, resulting in high economic burden, a major challenge for clinical treatment of RA [26]. Moxibustion is an external treatment based on the theory of Traditional Chinese medicine. It can not only stimulate the self-regulation function of the human body but is also widely used in the prevention, treatment, and alleviation of diseases because of its simple, inexpensive test and easy operation. Modern studies have also proved that moxibustion can stimulate the internal regulation ability of the body by affecting the function of immune cells, the level of cytokines and signal pathways, and other multilinks and multitargets [18, 27, 28]. Therefore, we choose the combination of moxibustion and drug therapy to explore a better treatment.

Traditional Chinese medicine believes that RA is mostly “deficient root and excessive superficial,” so in the study, we take “strengthening the body resistance to eliminate pathogenic factors” as the basic treatment principle, select two acupoints ST36 and BL23 to warm the yang and tonify the kidney, in order to strengthen the body resistance, and cooperate with local Ashi acupoints, warming the meridians and dredging collaterals to eliminate pathogenic factors. Our previous studies have proved that moxibustion ST36 and BL23 combined with ashi point can play an anti-inflammatory and analgesic role and can significantly relieve the clinical symptoms of patients [20, 21]. In addition, a large number of animal experiments also proved that moxibustion ST36 and BL23 could effectively reduce the proliferation of the synovial tissue and fibrous tissue in RA model, restore the balance between osteoblasts and osteoclasts, reduce inflammatory cytokines level, and thus improve the symptoms of joint swelling and pain [19, 29, 30].

The results of our study showed that, under standard treatment, the values of RAI, SJC, DAS28, and SF-MPQP in the two groups were significantly decreased, which objectively reflected that the symptoms of joint swelling and pain and disease activity of the patients were significantly improved, and the improvement degree in the treatment group with moxibustion was more significant. This result is basically consistent with our previous research results [20, 21], which all prove that moxibustion therapy combined with western medicine can improve the curative effect to a greater extent, improve the clinical symptoms of patients, and reduce the pain of patients.

### 4.2. Effects of Moxibustion on the Contents of CXCL1, *β*-EP, TNF*-α*, and IL-1*β* in RA Patients

Persistent synovitis is one of the characteristics of RA, which is mainly caused by the continuous migration of immune cells into the joints and the continuous production of various proinflammatory cytokines, of which chemokines, TNF-*α*, and IL-1*β* play a crucial role in synovitis [31]. First of all, chemotactic cytokines are one of the key factors for the migration of immune cells into the inflammatory synovium through the endothelial barrier [6]. CXCL1, as one of the chemokines, mainly attracts neutrophils and promotes the activity of inflammatory factors, which can be widely detected in the serum, synovial fluid, and synovial tissue of patients with RA. Second, in addition to the typical role of chemokines, CXCL1 also promotes angiogenesis because it contains N-terminal Glu-Leu-Arg (ELR) sequences [32]. It is worth noting that CXCL1 is not only associated with inflammation and angiogenesis but also has a significant correlation with neuropathic pain [33]. It is found that CXCL1 can act on peripheral neurons through its main receptor CXCR2, improve the sensitivity and excitability of peripheral sensory neurons, and cause peripheral neuron sensitization [10, 34]. On the other hand, inflammatory factors dominated by TNF-*α* and IL-1*β* can induce inflammation and stimulate RA synovium fibroblasts or macrophages to produce more chemokines [35]. In a series of studies, inhibition of TNF-*α* and IL-1*β* expression can reduce the expression of cytokines and chemokines, inhibit new synovial angiogenesis and cell proliferation, and thus alleviate the disease of RA patient [36–40].

In addition, *β*-EP, as an endogenous opioid peptide, has a strong analgesic effect on peripheral and central nervous system, including inflammatory pain. *β*-EP can reduce the excitability of nociceptors in RA patients, reduce the spread of action potential, and reduce the secretion of end inflammatory neuropeptides in central and peripheral nociceptors, so as to achieve analgesic effect [41]. In addition, *β*-EP synthesized and released by immune cells under inflammatory conditions can combine to corresponding receptors on the surface of immune cells and regulate inflammatory factors [42]. Studies have shown that *β*-EP can selectively inhibit the expression of IL-1*β* and TNF-*α* in synovium and foot inflammatory tissues of CIA rats [17]. This suggests that *β*-EP can ease inflammatory pain by inhibiting nerve excitation and regulating inflammation.

In our previous study, it was found that the contents of inflammatory factors dominated by IL-1*β* and TNF-*α* in serum of RA patients treated with moxibustion decreased significantly, which was also fully reflected in this study [20, 21]. It is worth noting that, in this study, the CXCL1 content in patient serum of the treatment group was significantly decreased, and it was significantly positively correlated with the improvement of IL-1*β* and TNF-*α*. The same results were not found in the control group. At the same time, the content of *β*-Ep in serum was significantly increased, correlation analysis showed that there was a negative correlation between the change of *β*-EP level and that of TNF-*a* and IL-1*β* level, but there was no significant difference (Table 3). Therefore, it is concluded that moxibustion may reduce level of CXCL1 by inhibiting the expression of TNF-*α* and IL-1*β*, thus inhibiting its recruitment of immune cell migration and proinflammatory effects, so as to achieve the effect of anti-inflammation and detumescence. In addition, the level of CXCL1 has been reduced, which means that the excitatory effect of peripheral neurons is inhibited, and hyperalgesia also has been improved. At the same time, the analgesic effect can also be achieved by moxibustion through upregulating the level of *β*-Ep in serum of RA patients. However, whether the anti-inflammatory effect of moxibustion is related to the regulation of *β*-EP level has not been fully confirmed in the study.

## 5. Conclusions

Moxibustion can improve the symptoms of joint swelling and pain in RA patients treated with conventional western medicine, which may be related to the influence of moxibustion on the levels of TNF-*α*, IL-1*β*, *β*-EP, and CXCL1. According to the results of our study, one of the effective mechanisms may be that moxibustion can reduce CXCL1 level by reducing the release of inflammatory factors and inhibit its effects of recruiting immune cells to migrate, promoting inflammation and causing pain. At the same time, the serum *β*-EP level was upregulated, and the analgesia and immune regulation were enhanced.

## Figures and Tables

**Table 1:** Clinical symptoms and routine inspection indexes.

Outcome measure		Treatment group (*n* = 30)	Control group (*n* = 31)	*P* value
Clinical symptoms				
RAI	Baseline	20.53 (11.45)	22.09 (18.69)	0.71^∇^
	Posttherapy	7.26 (6.46)	12.74 (12.11)	
	Change	13.50 (14.50)^*∗*^	6.00 (13.00)^*∗*^	0.04^∇^
SJC	Baseline	4.60 (3.39)	5.35 (4.36)	0.65^∇^
	Posttherapy	0.93 (1.38)	3.29 (3.58)	
	Change	4.00 (3.00)^*∗*^	2.00 (4.00)^*∗*^	0.02^∇^
DAS28	Baseline	5.33 (0.99)	5.50 (1.02)	0.49^Φ^
	Posttherapy	4.17 (0.94)	4.86 (1.40)	
	Change	1.19 (1.38)^*∗*^	0.69 (0.83)^*∗*^	0.04^∇^
SF-MPQ	Baseline	19.57 (3.71)	21.13 (10.78)	0.46^Φ^
	Posttherapy	10.10 (4.04)	19.26 (11.54)	
	Change	10.00 (7.00)^*∗*^	2.00 (4.00)^*∗*^	0.00^∇^

Routine inspection indexes				
ERS (mm/60 min)	Baseline	65.40 (34.90)	64.61 (33.77)	0.93^∇^
	Posttherapy	37.17 (27.42)	49.23 (29.55)	
	Change	24.50 (43.00)^*∗*^	12.00 (34.00)^*∗*^	0.07^∇^
CRP (mg/L)	Baseline	13.55 (14.79)^*∗*^	8.45 (29.07)^*∗*^	0.70^∇^
	Posttherapy	5.43 (7.61)^*∗*^	4.08 (10.66)^*∗*^	
	Change	7.63 (17.99)^*∗*^	2.7 (9.95)^*∗*^	0.56^∇^
RF (IU/ml)	Baseline	89.10 (350.95)^*∗*^	94.5 (285.80)^*∗*^	0.85^∇^
	Posttherapy	72.55 (109.53)^*∗*^	58.80 (207.80)^*∗*^	
	Change	10.95 (59.68)^*∗*^	18.00 (50.20)^*∗*^	0.27^∇^

Gaussian distribution values are represented as mean (SD), and non-Gaussian distribution values are represented as median (first quartile to third quartile)^*∗*^. ^Φ^*P* value by independent samples *t* test. ^*∇*^*P* value by Mann Whitney *U* test.				

**Table 2:** Changes of contents of CXCL1, *ß*-EP, TNF-*α*, and IL-1*β*.

Outcome measure	Treatment group (*n* = 30)	Control group (*n* = 31)	*P* value
CXCL1 (ng/mL)			
Baseline	2.65 (1.37)	2.81 (1.33)	0.57^∇^
Posttherapy	2.21 (1.22)	2.83 (1.66)	
Change	0.44 (0.79)	−0.01 (0.86)	0.03^Φ^
*β*-EP (pg/mg)			
Baseline	11.42 (6.07)	29.55 (14.66)	0.79^Φ^
Posttherapy	24.39 (11.76)	28.26 (15.02)	
Change	−2.43 (5.52)^*∗*^	−0.04 (4.09)^*∗*^	0.00^∇^
TNF-*α* (pg/mL)			
Baseline	25.41 (12.01)	27.29 (14.45)	0.58^Φ^
Posttherapy	20.04 (10.14)	25.45 (15.60)	
Change	3.45 (5.90)^*∗*^	1.55 (8.29)^*∗*^	0.04^∇^
IL-1*β* (pg/mL)			
Baseline	30.54 (13.97)	29.55 (14.66)	0.79^Φ^
Posttherapy	24.39 (11.76)	28.26 (15.02)	
Change	6.15 (8.65)	1.28 (8.51)	0.03^Φ^

Gaussian distribution values are represented as mean (SD), and non-Gaussian distribution values are represented as median (first quartile to third quartile)^∗^. ^Φ^*P* value by independent samples *t* test. ^*∇*^*P* value by Mann Whitney *U* test.			

**Table 3:** Correlation of *β*-EP and CXCL1 values with TNF-*α* and IL-1*β* values after treatment.

Outcome measure	Outcome measure	*r*	*P*
CXCL1	TNF-*α*	0.495	0.005
	IL-1*β*	0.758	<0.001

*β*-EP	TNF-*α*	−0.119	0.531
	IL-1*β*	−0.361	0.05

## Data Availability

The data used to support the findings of this study are available from the corresponding author upon reasonable request.
